# Polyneuropathy in Cerebrotendinous Xanthomatosis: Diagnostic Challenges and Potential for Therapeutic Intervention

**DOI:** 10.3390/brainsci14111159

**Published:** 2024-11-20

**Authors:** Antonio Edvan Camelo-Filho, Pedro Lucas Grangeiro Sá Barreto Lima, Francisco Luciano Honório Barreto Cavalcante, Oliver Reiks Miyajima, Carolina Figueiredo Santos, Rodrigo Fagundes da Rosa, André Luiz Santos Pessoa, Pedro Braga-Neto, Paulo Ribeiro Nóbrega

**Affiliations:** 1 Division of Neurology, Department of Clinical Medicine, Federal University of Ceará, Fortaleza 60430-372, Ceara, Brazil; edvan.camelo@gmail.com (A.E.C.-F.); pedro.lucas@alu.ufc.br (P.L.G.S.B.L.); francski2304@alu.ufc.br (F.L.H.B.C.); rodrigodarosa@alu.ufc.br (R.F.d.R.); andrepessoa10@yahoo.com.br (A.L.S.P.); paulo_r_med@yahoo.com.br (P.R.N.); 2 Center of Health Sciences, State University of Ceara, Fortaleza 60714-903, Ceara, Brazil; oliver.miyajima@aluno.uece.br; 3 Curso de Medicina, Universidade de Fortaleza, Fortaleza 60150-160, Ceara, Brazil; carolina.figsantos@gmail.com; 4 Division of Neuropediatrics, Hospital Infantil Albert Sabin, Fortaleza 60410-794, Ceara, Brazil; 5 Campus Parque Ecológico, Centro Universitário Christus, Fortaleza 60160-230, Ceara, Brazil

**Keywords:** cerebrotendineous xanthomatosis, polyneuropathy, EMG, nerve ultrasound

## Abstract

Cerebrotendinous xanthomatosis (CTX) is a rare metabolic disorder caused by mutations in the *CYP27A1* gene, leading to cholestanol accumulation in various tissues, including peripheral nerves. Polyneuropathy is an underrecognized feature with considerable variability in clinical presentation and neurophysiological findings in CTX. This review assesses the prevalence, clinical manifestations, and diagnostic methodologies of polyneuropathy in CTX, exploring its underlying mechanisms and potential treatment outcomes. A literature review was conducted using PubMed, Embase, and the Virtual Health Library databases with search terms related to CTX and polyneuropathy. A total of 892 articles were initially identified, with 59 selected for in-depth analysis. The review focused on studies examining peripheral nerve involvement in CTX, including nerve conduction studies, electromyography, and nerve ultrasound. Polyneuropathy in CTX was observed in 50% to 77.7% of patients across multiple case series. Neurophysiological findings varied, with reports of axonal, demyelinating, and mixed polyneuropathies. Clinical presentation included lower limb atrophy, pes cavus, and distal weakness, with sensory symptoms less frequently reported. Treatment with chenodeoxycholic acid (CDCA) showed potential in improving nerve conduction parameters, although the response was variable and dependent on the timing of intervention. Polyneuropathy in CTX presents significant diagnostic challenges due to its heterogeneous presentation and varying neurophysiological findings. Early recognition and intervention are crucial for improving patient outcomes. Peripheral nerve ultrasound is a promising diagnostic tool, complementing traditional neurophysiological assessments. Further research is needed to standardize protocols and explore the full therapeutic potential of CDCA in managing CTX-related polyneuropathy.

## 1. Introduction

Cerebrotendinous xanthomatosis (CTX) is a rare autosomal recessive lipid storage disorder caused by pathogenic variants in the *CYP27A1* gene, which encodes sterol 27-hydroxylase, an enzyme in the cytochrome P450 oxidase family [1,2]. This enzyme converts cholesterol to bile acids, and its compromise leads to decreased bile acid synthesis and excess production of cholestanol, which accumulates in body tissues, particularly in tendons, eye, and peripheral and central nervous system [1,2].

CTX has been recently recognized as a considerably underdiagnosed disease, a fact that might be related to a highly heterogeneous clinical presentation with a wide range of symptoms, severity, and age of onset [3,4]. The clinical presentation includes neonatal jaundice or cholestasis, persistent diarrhea, juvenile cataracts, tendon xanthomas, osteoporosis, coronary heart disease, progressive neuropsychiatric issues such as intellectual disability or dementia, psychiatric symptoms, pyramidal and cerebellar signs, progressive myelopathy, extrapyramidal symptoms, seizures, and peripheral neuropathy [1,2,3,4,5,6,7].

Polyneuropathy is a frequent neurological condition characterized by peripheral nerve damage that may impact motor, sensory, or autonomic nerve fibers. The condition affects approximately 1–3% of the general population, with the prevalence rising to about 5.5% in individuals over the age of 55 [8]. Electrodiagnostic testing further classifies polyneuropathy as primarily axonal or demyelinating, affecting motor, sensory, or both types of fibers [9].

Peripheral neuropathy is a frequently reported and disabling feature of CTX [10]. Nerve conduction studies (NCS) and electromyography (EMG) may reveal demyelinating, axonal, or mixed polyneuropathy. Signs and symptoms of polyneuropathy are often subtle or difficult to detect because central nervous system and psychiatric involvement may overshadow the clinical presentation [11]. The severity of polyneuropathy varies significantly among patients, ranging from asymptomatic to severe cases [6,11,12]. Assessing the peripheral nervous system plays a crucial role in the diagnosis and monitoring of CTX.

This review aims to provide a comprehensive overview of polyneuropathy in CTX.

The objective is to explore the prevalence of polyneuropathy in individuals with this condition and characterize its clinical manifestations. To date, this is the first systematic review to explore emerging diagnostic technologies, such as nerve ultrasound and small fiber assessment, alongside traditional methods like NCS/EMG, addressing gaps in current diagnostic practices and proposing more sensitive tools for evaluating polyneuropathy in CTX patients. Finally, the potential for treatment to improve polyneuropathic symptoms will be discussed, highlighting advancements and challenges in the management of this rare but impactful condition.

## 2. Materials and Methods

We performed a systematic review on PubMed, Embase, and the Virtual Health Library databases. The initial search was conducted in June 2024 using the following search terms: “cerebrotendinous xanthomatosis”, “polyneuropathy”, “peripheral nerve”, “neurophysiological study”, “electroneuromyography”, ”small fiber neuropathy”, and “nerve ultrasound”. All articles found in the search were considered for inclusion. We also screened the articles’ reference lists for possible relevant publications.

This systematic review was conducted according to the Preferred Reporting Items for Systematic Reviews and Meta-analyses (PRISMA) reporting guideline [13].

We included studies with patients diagnosed with CTX who developed peripheral neuropathy. There was no restriction regarding age, previous diseases, gender, or ethnicity.

Criteria for inclusion were: (1) Articles regarding peripheral neuropathy in CTX; (2) CTX confirmed by molecular or genetic testing; (3) Articles involving only human subjects. Exclusion criteria were: (1) Reviews or Conference Abstracts; (2) Articles written in languages other than English; (3) Articles that did not include any case evaluation for peripheral neuropathy.

**Figure 1:**
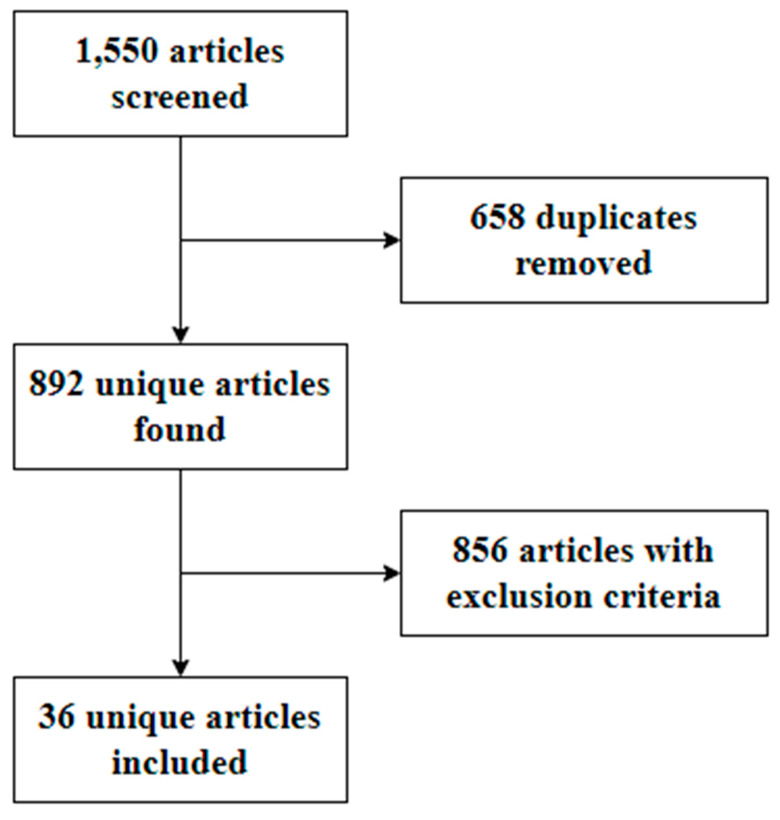
Flowchart showing the study selection for review.

Two independent evaluators initially screened the studies from their titles and abstracts. A third reviewer made the final decision in cases of discordance. Full texts of the resulting studies were evaluated according to eligibility criteria. Study selection results were reported using a PRISMA Flowchart (Figure 1).

A standardized form was used for data extraction from included studies. Information extracted included country, author, year, objectives, study design, overall prevalence of peripheral neuropathy, prevalence of each form of neurological impairment, median age of subjects, sample size and type, time of evaluation from disease onset, and the instruments used for diagnosis.

The primary search in Pubmed, Embase, and the Virtual Health Library yielded 892 articles. The records identified were then screened and selected by two independent authors, excluding articles that did not assess peripheral nerve compromise in patients with cerebrotendinous xanthomatosis. After duplicate removal and exclusion of articles not considered relevant for the description of the CTX peripheral nerve profile, 36 publications were included and had their reference lists analyzed for additional data review.

## 3. Results

### 3.1. Prevalence of Neuropathy in CTX

Neuropathy is a typical characteristic of various forms of cerebellar ataxias, both hereditary and sporadic [14]. When investigating cerebellar ataxias, the pattern of peripheral nerve involvement and the accompanying clinical symptoms can be very helpful in narrowing down the etiologic diagnosis, such as in CTX [15]. When diagnosing a patient with cerebellar ataxia and neuropathy, a thorough medical history, an understanding of disease progression, and an accurate family history are all necessary. Features of neuroimaging and the results of neurophysiology tests also play crucial roles in diagnosing and treating these conditions [16,17].

Polyneuropathy is a frequently observed feature of CTX, with multiple studies indicating a prevalence ranging from 50 to 77.7% [3,6,10,11,15,18,19,20,21,22]. Table 1 summarizes the results of the review of selected CTX populations of five international cohorts. We analyzed 164 patients from Europe (Italy, The Netherlands, Spain), South America (Brazil), and Asia (China, Republic of Korea, India) [3,10,11,15,18,19].

Patients experienced a significant delay in diagnosis, with CTX typically being identified in the late third decade of life (between 27.6 and 38.5 years), despite symptoms usually beginning around 11.2 years of age [3]. In most cases, polyneuropathy in CTX was associated with other clinical features, with 76.9% of CTX patients with polyneuropathy also reporting cognitive impairment, and 61.5% referring to psychiatric disturbances in one case series [10,23].

### 3.2. Clinical Features

In a study by Pilo-de-la-fuente et al., all eight patients with peripheral neuropathy had mild to moderate clinical manifestations consisting of lower limb calf atrophy, distal weakness, and pes cavus, but none had sensory symptoms [11]. Conversely, another case series [18] showed that only four out of the twenty patients with documented polyneuropathy reported neuropathic sensory symptoms. This delay in presentation, in some cases, might be related to cognitive dysfunction and a lack of symptom perception.

**Figure 2:**
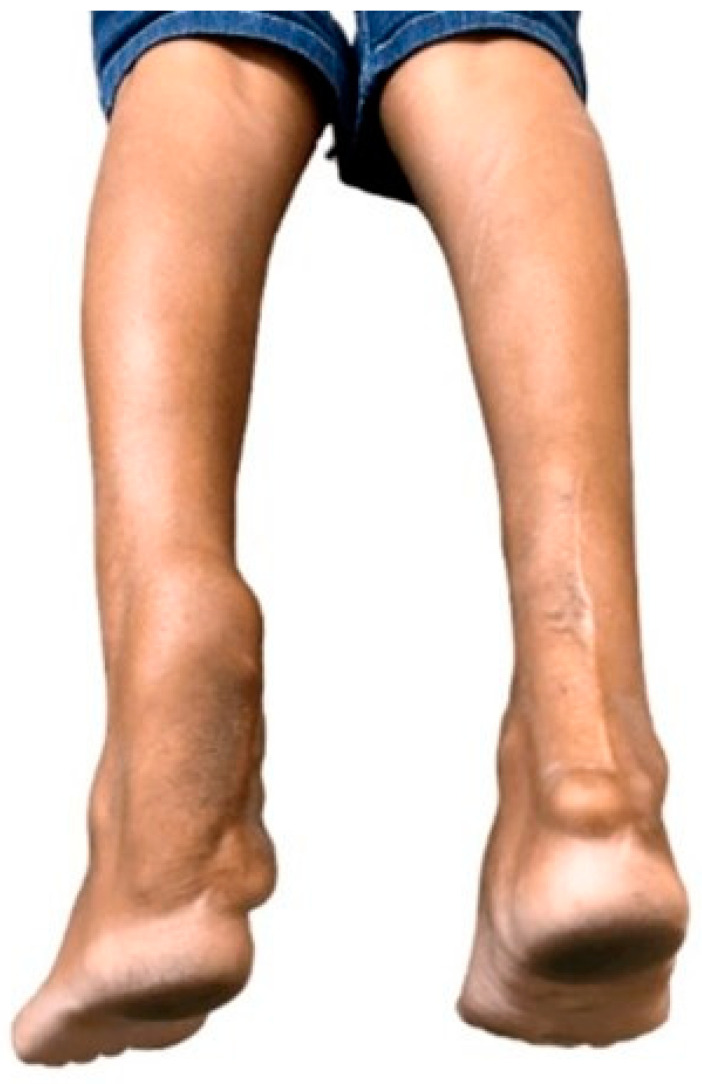
Tendon xanthoma in CTX.

The prevalence of pes cavus in CTX polyneuropathy ranges from 34.6% to 88.5% [3,6,10,11,15,18,19]. Patients might not report any neuropathic symptoms and may only present to the clinic in the later stages of the disease, characterized by tendon xanthomas (Figure 2), pes cavus, and lower limb atrophy, in addition to other general neurological features [6]. CTX must remain a differential diagnosis in patients with pes cavus and suspected hereditary neuropathy. The absence of xanthomas should not be considered as an isolated clinical marker for exclusion of CTX, as it may not be found even in the presence of polyneuropathy [11,15,19].

Moreover, no specific scale is designed for evaluating polyneuropathy in the CTX population, and most studies do not adequately address this issue. The diagnosis of polyneuropathy has predominantly relied on NCS and EMG evaluations, often without incorporating formal clinical assessments or specific scales for peripheral neuropathy [10,18,19,22,24,25,26,27]. This approach, particularly for follow-up and early detection of clinical progression, may overlook important aspects of the condition.

### 3.3. Neurophysiological Findings

Peripheral neuropathy is a recognized feature of CTX; however, the precise mechanisms underlying its development—whether predominantly attributable to demyelination or axonal degeneration—remains a topic of ongoing debate. Neurophysiological studies conducted across different CTX cohorts have shown varying results [10,18,19,24,25,26,28], which contributes to the complexity of understanding the pathology. Some studies have documented it as a primarily length-dependent axonal polyneuropathy [29,30,31], while others have identified demyelination as the dominant feature. This discrepancy has led to continued debate within the field [32].

Based on NCS and EMG studies, Verrips et al., Ginanneschi et al., and Chen et al. all reported findings that support axonal involvement as a significant feature of peripheral neuropathy in CTX [18,19,25]. Verrips et al. identified predominantly axonal neuropathy in their cohort, with EMG findings confirming this pattern in seven out of ten patients [19]. Similarly, Ginanneschi et al. found that 76.9% of their patients exhibited predominantly axonal polyneuropathy [18]. This finding is further supported by Chen et al., who observed axonal sensory-motor polyneuropathy in three out of four CTX cases [25]. Also, nerve biopsy findings from the studies by Pop et al. (1984), Donaghy et al. (1990), Verrips et al. (2000), and Chen et al. (2011) highlight the presence of axonal degeneration as a key feature of the neuropathy associated with CTX [18,19,25,33,34].

In contrast, several other case series identified a primary demyelinating polyneuropathy from 56% up to 100% of their cohorts [3,11,15,28,32,35,36,37,38,39,40]. Studies suggest that CTX patients often exhibit features of demyelination, possibly related to the toxic effects of cholestanol on Schwann cells, which maintain and produce the myelin sheath. This can be reflected in the slowing of nerve conduction velocities seen in electrodiagnostic studies, a hallmark of demyelinating polyneuropathy [26]. Pilo et al. suggest that polyneuropathy in CTX is primarily demyelinating. In their study, they found that polyneuropathy was predominantly sensorimotor and demyelinating in 62% of patients. Fussiger et al., also described CTX neuropathy as primarily demyelinating in all patients with polyneuropathy from their cohort [10]. Ohnishi et al. conducted a study that provided histological evidence of significant de-and remyelination and onion bulb formation in the sural nerve biopsies of CTX patients, suggesting a primary involvement of the myelin sheath and Schwann cells [41]. Wang et. al described a patient with severe demyelinating polyneuropathy who underwent a nerve biopsy which showed a marked reduction in myelinated fibers, particularly those of larger diameters, and the presence of onion bulb formations without evidence of axonal degeneration [6].

Additionally, mixed axonal-demyelinating polyneuropathy was reported in several cases [10,11,15,19,25,36,42]. These findings highlight the heterogeneity of peripheral nerve involvement in CTX, with some patients exhibiting purely axonal, purely demyelinating, or mixed forms of polyneuropathy.

To better address this issue, Wang and colleagues proposed a classification system for CTX-related polyneuropathy, categorizing it into three pathological types: (i) axonal polyneuropathy, characterized by axonal degeneration and regeneration clusters; (ii) demyelinating polyneuropathy, marked by extensive demyelination, thin myelin sheaths, and onion bulb formations; and (iii) mixed polyneuropathy, where both axonal and myelin damage are equally involved [6]. The literature indicates that possibly the pathology of polyneuropathy in CTX likely involves both demyelination and axonal degeneration. This variability among patients may be related to individual differences in disease progression or the timing of neurophysiological assessments. The pathological findings from nerve biopsies also reflect this heterogeneity. For example, Pilo et al. found that in their series, 62% of patients presented with sensorimotor and demyelinating polyneuropathy, though axonal and mixed neuropathies were also observed [4,10]. Similarly, Verrips et al. found that sural nerve biopsies in three CTX patients showed decreased densities of large-diameter myelinated fibers, indicative of axonal degeneration and atrophy. However, one patient exhibited markedly different morphology, with both large and small myelinated and non-myelinated fibers severely decreased and the presence of large Schwann cell clusters resembling onion bulbs, primarily in the proximal part of the biopsy [19]. This could suggest that CTX polyneuropathy may be a primary proximal demyelinating process with secondary distal axonal degeneration.

This debate regarding the underlying pathology of CTX-related polyneuropathy may be further complicated by the variability of findings in nerve conduction studies. Another important consideration is the variability in how neurophysiological studies, such as NCS and EMG, are conducted and interpreted [15]. This variability may contribute to the discrepancies seen across studies. In severe cases, primary demyelination may lead to secondary axonal loss, which could be misinterpreted as purely axonal degeneration during electrodiagnostic assessments [15]. Moreover, recent studies using nerve ultrasound have pointed out the presence of heterogeneous nerve enlargement in proximal nerves in CTX patients, a finding typically described in demyelinating polyneuropathies [43]. This finding could further explain the mixed presentations of polyneuropathy.

The involvement of the central nervous system (CNS) in CTX further supports the possibility of a primary demyelinating origin for polyneuropathy. Histological examination of the CNS in CTX patients has shown lipid depositions, with demyelination and gliosis observed in affected areas [44]. This CNS involvement raises the hypothesis that a similar demyelinating process could occur in peripheral nerves, although further studies are needed to confirm this link.

Despite these controversies, this doubtful clinical scenario warrants further investigation to understand the mechanisms behind the variability in peripheral nerve involvement and whether changes in disease management or diagnostic criteria are influencing these findings. Further research, including larger sample sizes and multicentric studies, is necessary to consolidate these observations and clarify the pathophysiology of CTX-related polyneuropathy.

In conclusion, the development of peripheral neuropathy in CTX appears to result from both demyelination and axonal degeneration [6,41]. These changes are likely secondary to the accumulation of abnormal lipids in neural tissues, which disrupts the integrity of nerve fibers. The combination of demyelination and axonal degeneration can lead to more severe and progressive forms of polyneuropathy, underscoring the need for more comprehensive studies to resolve these discrepancies in clinical and neurophysiological findings.

### 3.4. Small Fiber Neuropathy

Small fiber neuropathy (SFN) is recognized as the compromise of small somatic sensory and autonomic C fibers, which are responsible for somatic and autonomic functions, respectively. Patients with SFN often experience symptoms such as pain, burning, tingling, numbness, and cardiac and other autonomic symptoms, which can significantly affect the quality of life due to the combination of neuropathic pain and autonomic dysfunction [45,46].

SFN can arise from various conditions, including diabetes, Sjögren’s syndrome, lupus, and light-chain amyloidosis. Additionally, hereditary neuropathies such as transthyretin-related amyloidosis and Fabry disease are also known to cause SFN [47]. In CTX, SFN evaluation has been infrequently performed. Arpa et al. [48] reported a case of postganglionic cholinergic failure in a patient with peripheral neuropathy, identified through sympathetic skin response testing. In contrast, another case report revealed a patient with severe polyneuropathy who exhibited no significant small fiber impairment, as assessed by cardiovascular reflex quantification, spectral analysis of heart rate variation, and sympathetic skin responses [49]. The authors proposed that CTX predominantly affects large nerve fibers. Lastly, a more comprehensive evaluation was conducted in a case series of four Chinese patients [25]. In addition to analyzing R-R interval variation and sympathetic skin responses, intraepidermal nerve fiber (IENF) density measurements were performed in four patients with polyneuropathy, revealing a high incidence (75%) of small fiber involvement [25]. These discrepant results may be related to the techniques used in the studies, as IENF measurement is the gold standard for SFN diagnosis and were not performed in previous studies [50]. Lastly, SFN may represent an early stage of disease presentation, but little is known in that population. Further studies are needed to assess SFN involvement in CTX patients using standardized techniques and a comprehensive electrophysiological workup specifically directed at SFN.

### 3.5. Nerve Ultrasound

Peripheral nerve ultrasound is a valuable diagnostic and monitoring tool for neuropathies [51], yet its application in CTX remains underexplored. This imaging technique complements clinical assessments and electrodiagnostic studies by providing crucial structural information that aids in diagnosing and identifying pathological changes. Nerve ultrasound allows for real-time imaging of peripheral nerve structures, enabling the detection of atrophy, thickening, and other structural abnormalities, which is particularly beneficial in identifying demyelinating or deposit polyneuropathies, such as seen in CTX.

Nerve ultrasound has proven especially sensitive for detecting mild neuropathies and can enhance diagnostic accuracy when combined with NCS. In contrast, NCS remains the preferred method for assessing the functional properties of peripheral nerves, providing key data on conduction velocities and amplitudes that help distinguish between axonal and demyelinating conditions. Loewenbrück et al. compared the diagnostic accuracy of nerve ultrasound and NCS in hereditary and sporadic non-entrapment neuropathies. This study found that for acquired non-entrapment peripheral neuropathies, NCS had superior sensitivity (97%) compared to ultrasound (US) (56%), while both methods had comparable specificity (NCS 89%, US 93%) [52].

Overall, the evidence supports the use of nerve ultrasound as a complementary diagnostic modality in hereditary polyneuropathies like CTX, providing morphological insights that can enhance clinical evaluation and management. Specific nerve size and fascicle echogenicity-related ultrasound patterns have been documented in other hereditary neuropathies such as Charcot-Marie-Tooth disease, Fabry disease, hereditary neuropathy with liability to pressure palsies (HNPP), transthyretin amyloidosis, autosomal dominant spinocerebellar ataxias (SCA), cerebellar ataxia with neuropathy and vestibular areflexia syndrome (CANVAS), Friedreich’s ataxia, and minifascicular neuropathy [51,53,54,55,56].

Yoon et al. [57] were the first to report the ultrasonographic findings of peripheral nerves in a patient with CTX. The patient presented with foot hypoesthesia and bilateral Achilles tendon xanthomas, and NCS/EMG revealed sensory-motor demyelinating polyneuropathy. US demonstrated diffusely enlarged peripheral nerves, including the ulnar, median, tibial, and fibular nerves, and the C5–C7 nerve roots. Roeben et al. [43] further characterized peripheral nerve morphology in a case series of four patients. This study revealed mild to moderate hypoechogenic thickening of sensorimotor nerves in all four patients and mild to moderate enlargement of pure sensory nerves and cervical roots in some of the patients. The study also suggested the presence of inhomogeneous nerve enlargement. Both authors [43,57] hypothesized that nerve enlargement in CTX might result from the accumulation of abnormal lipids, such as cholestanol, within peripheral nerves. Additionally, thickening of the nerve roots and trunks of the lumbosacral plexus or cauda equina has also been observed on magnetic resonance imaging (MRI) sequences [58].

The correlation between nerve ultrasound enlargement and disability in other hereditary polyneuropathies is supported by several studies. Zanette et al. demonstrated that in CMT1A, nerve cross-sectional area (CSA) measured by ultrasound is significantly associated with clinical severity scores, such as the CMT Neuropathy Score version 2 (CMTNS2) and its examination subscore (CMTES2). Their multivariate analysis indicated that median forearm CSA significantly influenced these clinical scores, suggesting that nerve size could serve as a biomarker for disease severity and progression [1]. Yiu et al. found that in pediatric CMT1A, nerve CSA was significantly increased compared to controls and correlated with neurologic disability [59]. This study highlighted the potential of peripheral nerve ultrasound as a diagnostic tool and an outcome measure in clinical trials for CMT1A [2]. Lastly, Noto et al. also reported that in CMT1A, there is a positive correlation between the CMT Neuropathy Score and the CSA of the median and great auricular nerves, further supporting the relationship between nerve enlargement and disease severity [3,60]. Therefore, this technique may be useful in assessing disease progression and severity in CTX. In summary, nerve ultrasound shows potential as a biomarker in CTX by measuring nerve enlargement. Further research is needed to establish nerve size correlation with clinical outcomes in CTX, as has been established for CMT1A, and its role in guiding treatment decisions.

The accessibility of diagnostic tools such as nerve ultrasound in resource-limited settings is shaped by several challenges, including the costs of equipment, the need for specialized training, maintenance difficulties, and uneven distribution of resources [61,62,63,64,65,66,67]. In resource-limited settings, the choice to use nerve ultrasound alongside NCS and EMG for peripheral neuropathies should be based on the clinical context, resource availability, and the expertise of healthcare providers [62,63,64,65]. Point-of-care ultrasound (POCUS) has demonstrated its value as a cost-effective and repeatable diagnostic tool, especially in rural and underserved areas [64,65]. For instance, some studies report that the availability of ultrasound in primary care facilities in low- and middle-income countries (LMICs) is as low as 1.2%. However, compared to MRI, ultrasound is a less expensive option, as highlighted by studies showing cost savings in various diagnostic scenarios. For example, a pediatric contrast-enhanced ultrasound (CEUS) study revealed significant savings, with CEUS costing USD94 per exam versus USD274 for MRI [66]. Another study found that ultrasound was the least expensive method for evaluating symptomatic full-thickness supraspinatus tendon tears, with MRI having a higher incremental cost-effectiveness ratio (ICER) [67]. Ultrasound’s lower cost, real-time imaging capabilities, and avoidance of sedation or ionizing radiation make it an invaluable tool for diagnosing and managing hereditary neuropathies like CTX.

### 3.6. New Technologies

Lee (2022) utilized tractography and diffusion kurtosis imaging (DKI) to assess CNS neuroimaging changes in CTX patients who had discontinued CDCA treatment for three years [68]. The findings revealed significant fiber loss in brain tissue, despite serial conventional MRI studies showing no substantial changes in cerebral white matter. The DKI results indicate complex pathophysiological alterations in CTX, encompassing both axonal loss and demyelination. These findings are initial but promising, highlighting the need for further studies to better correlate these neuroimaging changes with clinical outcomes in CTX.

Artificial intelligence (AI) has also demonstrated considerable potential in the diagnosis of neuromuscular disorders [69,70,71,72,73]. By integrating AI into diagnostic processes, machine and deep learning algorithms are being used to enhance the accuracy and efficiency of traditional diagnostic tools such as NCS/EMG. AI models have shown high diagnostic accuracy in distinguishing between normal and pathological EMG signals, with reported accuracies ranging from 67% to 99.5% for conditions like amyotrophic lateral sclerosis and myopathy [71]. In the context of peripheral neuropathies, AI has been effectively utilized in corneal confocal microscopy to diagnose diabetic neuropathy [70]. Given the variability in clinical presentation of CTX, such innovative tools could prove invaluable in enhancing early diagnosis and management [69]. However, for AI models to be reliable, they require large and diverse datasets for validation. Innovative imaging and AI hold promise for improving diagnosis and treatment of CTX and related neuropathies, but further research is needed to confirm their effectiveness.

### 3.7. Treatment

CTX is a metabolic disorder caused by pathogenic variants in the *CYP27A1* gene, which encodes the sterol 27-hydroxylase enzyme. This enzymatic defect disrupts cholesterol metabolism, leading to the accumulation of precursors such as cholestanol, which can be up to 10 times higher than normal levels, despite cholesterol levels being normal or low [74]. The most promising treatment for CTX currently is chenodeoxycholic acid (CDCA), a bile acid that helps reverse the biochemical abnormalities associated with the disease. CDCA works by inhibiting the classic cholesterol metabolism pathway through negative feedback on the 7α-hydroxylase enzyme, reducing abnormal bile acid synthesis [1]. Research has shown that early intervention with CDCA can significantly improve prognosis, especially if therapy begins before significant neurological symptoms arise.

Berginer’s groundbreaking research in the early 1980s demonstrated that treatment with CDCA led to the normalization or significant reduction in cholestanol levels, which in turn resulted in notable improvements in organ function and long-lasting clinical benefits for patients with CTX [75,76,77].

A pivotal 1984 study showed that CDCA induced a threefold reduction in plasma cholestanol levels, leading to the resolution or significant improvement of clinical symptoms in most of the 17 CTX patients. Notably, dementia improved in 10 out of 13 patients, pyramidal signs diminished or disappeared in 13 out of 17, cerebellar dysfunction cleared in 3 patients and improved significantly in 9, neuropathy improved in 6, electroencephalograms improved in 8, and an increase in white matter was observed in 7 patients [76].

A decade later, similar outcomes were reported in a follow-up study involving 13 CTX patients [78]. Following Berginer’s research, other studies further corroborated the effectiveness of CDCA in lowering cholestanol levels and improving organ function. For instance, Mignarri et al. (2016) reported sustained reductions in biomarkers and stable disability scores over four years in 12 out of 19 CTX patients [79]. In a comprehensive review of 43 CTX cases treated with CDCA, Duell and colleagues found a 57% improvement rate in clinical symptoms, accompanied by a reduction in plasma cholestanol levels to an average of 6 mg/L [34,74].

Two single-center retrospective cohort studies (total N = 63) conducted by Verrips et al. confirmed significant improvements in biomarker levels, such as serum cholestanol and 7αC4, along with the resolution, stabilization, or improvement of clinical signs in both children and adults. In a smaller study involving 14 children and adults (ages 8–59), who received long-term CDCA treatment for a mean period of five years, del Mar Amador et al. observed that clinical improvement was mostly limited to younger patients (aged ≤ 25 years) who had received treatment within fifteen years of neurological symptom onset. Similarly, another retrospective study in Dutch patients with CTX (N = 56) showed that patients treated before the age of 24 experienced a complete resolution of neurological symptoms, whereas 61% of those treated after age 24 experienced a worsening of symptoms, with parkinsonism being particularly resistant to treatment [80]. These studies underscore the importance of early diagnosis and intervention in CTX management.

Recently, a phase 3, randomized, placebo-controlled, double-blind, crossover study (RESTORE) involved fourteen adult participants showed that CDCA withdrawal led to a significant increase in biomarkers, including a 20-fold rise in urinary 23S-pentol and a 2.8-fold decrease in plasma cholestanol, alongside heightened levels of 7αC4. CDCA was generally well-tolerated, with diarrhea and headache being the most common adverse effects. These findings highlight the critical role of CDCA in stabilizing CTX biomarkers and preventing disease progression [81].

Another potential treatment explored is the use of HMG-CoA inhibitors [1]. The clinical and metabolic outcomes of this treatment have shown mixed results across different studies [82,83,84,85,86]. Some research has indicated beneficial effects of statins, including reductions in cholestanol, cholesterol, alongside improvements in lipoprotein and cholesterol metabolism. In certain studies, clinical improvements were observed when statins were combined with CDCA therapy [83,84]. However, other studies have found that statins did not effectively reduce abnormal bile acid production or stabilize symptoms [82,85]. No clinical trials have been conducted to evaluate the use of statins in CTX, and there are no studies assessing the effect of statins on CTX neuropathy. Although some authors support combining statins with CDCA to potentially improve or stabilize patient outcomes, a clear consensus on this approach has not yet been established [87].

### 3.8. Follow-Up Assessment

There is limited information on neurophysiological studies conducted during follow-up to assess the response to treatment in the peripheral nerve. Berginer et al. presented a case series of seven patients with peripheral neuropathy who had been using CDCA for at least 1 year [76]. The authors concluded that all patients showed improvement in sensory loss during physical examination, but NCS/EMG assessments were not reported.

Donaghy et al. [34] reported the first case of a CDCA-treated CTX patient who underwent electrophysiological follow-up assessment. The patient received CDCA treatment during two separate periods of 10 and 6 months, which resulted in improvement in sensory symptoms and increased motor and sensory nerve conduction velocities. However, CDCA was discontinued within the first two years, and nerve conduction deteriorated to pretreatment levels after the interruption [34]. Tokimura et al. [24] presented a case series of seven patients, two of whom had polyneuropathy and received oral CDCA for 1 year. The patients did not show significant changes, and the authors hypothesized that there was irreversible peripheral nerve damage, suggesting that such patients should be treated in the early stages of the disease [24]. Another case series of CTX patients [18] monitored 20 patients with polyneuropathy who were available for electrophysiological follow-up. They found that CDCA treatment improved electrophysiological conduction parameters, regardless of the duration of therapy. The improvement was mainly observed in nerve conduction velocities, while nerve amplitudes remained unchanged [18]. They believed that CDCA treatment did not influence the regeneration of axons but increased the conduction of still-excitable fibers, suggesting that CDCA treatment could promote myelin synthesis in nerve fibers with residual unaffected axons. Notably, only 4 out of the 20 patients reported neuropathic sensory symptoms, which remained unchanged after CDCA treatment [18].

Pilo et al. reported on six patients who underwent neurophysiological studies during follow-up to assess their response to treatment [10]. Two of the patients continued to deteriorate despite receiving treatment CDCA and statin for 6 years. Two patients showed stabilization 2 years after initiating treatment (one treated exclusively with CDCA and the other with the combination of CDCA and a statin). In the remaining 2 cases, neurophysiological improvement was observed following treatment with CDCA and a statin, with improvements in amplitudes and nerve conduction velocities noted after one and three years [10]. However, one of these patients continued to experience progressive ataxia, paraparesis, and cognitive decline despite improvements in peripheral nerve function. Mondelli et al. (2001) reported the follow-up of five CTX patients treated for 11 years with CDCA. Three patients presented with a baseline demyelinating polyneuropathy characterized by reduced motor conduction velocity (MCV) [32]. After 4 months of therapy with CDCA, MCV normalized in these three patients and did not show any significant change over the 11-year observation period. The authors suggest that CDCA therapy should be initiated as early as possible before axonal damage is present and therefore irreversible. Also, the study by Amador et al. (2018) investigated the effects of CDCA treatment in patients CTX, focusing on clinical, neurophysiological, and brain structural outcomes [28]. The study found that CDCA treatment led to significant clinical improvement in patients who began treatment early, particularly those under 25 years old and within 15 years of symptom onset. There was a significant improvement in electrophysiological parameters of peripheral neuropathy, which was predominantly length-dependent. This suggests that CDCA may enhance nerve conduction, potentially by promoting myelin synthesis in fibers with residual axons. The improvement in nerve conduction velocities under CDCA treatment suggests a beneficial effect on demyelinating components, while the stabilization of clinical symptoms may reflect broader neuroprotective effects.

Cholestanol levels are a well-recognized biomarker for diagnosing and monitoring CTX. Elevated cholestanol in plasma is a key indicator of CTX, resulting from *CYP27A1* pathogenic variants that impair bile acid synthesis and lead to cholestanol buildup. Measuring cholestanol is essential for both initial diagnosis and assessing treatment response. In individuals with CTX, cholestanol levels are notably higher than normal, and treatment with CDCA can lower these levels, often aligning with clinical improvement. Berginer et al. demonstrated that CDCA therapy normalized cholestanol levels and helped prevent disease progression, while Duell et al. found that CDCA significantly reduced plasma cholestanol, with 63% of patients achieving optimal low levels [74,76]. Mondelli et al. (2001) studied five patients with cerebrotendinous xanthomatosis (CTX) who underwent treatment with chenodeoxycholic acid (CDCA) over an 11-year period [32]. The researchers observed a significant reduction in cholestanol levels, with levels reaching the normal range, which is a primary goal of CDCA therapy in CTX management. However, certain studies indicate that cholestanol may not always be sufficient for diagnosing atypical cases where levels may be within or close to the normal range. In such situations, supplementary markers, including neuroimaging, genetic evaluation, bile acid precursors, and bile alcohols dosing, may provide additional diagnostic value.

## 4. Conclusions

Polyneuropathy in cerebrotendinous xanthomatosis (CTX) presents significant diagnostic challenges, primarily due to its variable clinical manifestations and the ongoing debate regarding its underlying pathological mechanisms. While some studies suggest length-dependent axonal neuropathy as the predominant feature, others emphasize prominent demyelination, indicating a need for more comprehensive and standardized approaches to diagnosis and classification. This complexity highlights the need for standardized protocols and larger, multicentric studies to better understand CTX’s neuropathic profile.

Given that CTX is a treatable condition, early and accurate diagnosis of polyneuropathy is crucial for improving patient outcomes, particularly in cases presenting with lower limb atrophy, pes cavus, and xanthomas as an initial scenario. Early recognition and intervention can significantly influence the disease trajectory and enhance the quality of life for affected individuals. Peripheral nerve ultrasound emerges as a promising diagnostic tool, offering valuable structural insights that could facilitate earlier intervention and provide biomarkers for neurological damage. Continued research into the neurophysiological and imaging characteristics of CTX-related neuropathy will be essential in refining therapeutic strategies and improving the quality of life for affected individuals.

## Figures and Tables

**Table 1:** Peripheral nerve findings in CTX patients.

	Verrips et al. (2000) [19]	Pilo et al. (2011) [10]	Ginanneschi et al. (2012) [18]	Zhang et al. (2020) [15]	Fussiger et al. (2024) [3]
Number of patients	10	25	35	21	38
Country	The Netherlands	Spain	Italy	China	Brazil
Age at diagnosis (mean)	38.5	39	32.8	28.5	27.6
Cerebellar signs	60%	76%	51.4%	80.9%	48.6%
Cognitive impaitment	-	80%	-	71.4%	80.5%
Polyneuropathy	70%	64%	74.2%	76.1%	50%
EMG ^1^					
(I) Axonal	85.7%	37.5%	76.9%	25%	0%
(II) Demyelinating	-	50%	23.1%	56%	100%
(III) Mixed neuropathy	14.3%	12.5%	-	19%	-

^1^ EMG: Electroneuromyography.

## Data Availability

No new data were created or analyzed in this study.
